# Analysis of Enhanced Current-Generating Mechanism of *Geobacter sulfurreducens* Strain via Model-Driven Metabolism Simulation

**DOI:** 10.1371/journal.pone.0073907

**Published:** 2013-09-13

**Authors:** Jing Meng, Zixiang Xu, Jing Guo, Yunxia Yue, Xiao Sun

**Affiliations:** 1 State Key Laboratory of Bioelectronics, Southeast University, Nanjing, China; 2 Key Laboratory of Systems Microbial Biotechnology, Tianjin Institute of Industrial Biotechnology, Chinese Academy of Sciences, Tianjin, China; Texas A&M University, United States of America

## Abstract

Microbial fuel cells (MFCs) are a class of ideal technologies that function via anaerobic respiration of electricigens, which bring current generation and environmental restoration together. An in-depth understanding of microbial metabolism is of great importance in engineering microbes to further improve their respiration. We employed flux balance analysis and selected Fe(iii) as a substitute for the electrode to simulate current-generating metabolism of *Geobacter sulfurreducens* PCA with a fixed acetate uptake rate. Simulation results indicated the fluxes of reactions directing acetate towards dissimilation to generate electrons increased under the suboptimal growth condition, resulting in an increase in the respiration rate and a decrease in the growth rate. The results revealed the competitive relationship between oxidative respiration and cell growth during the metabolism of microbe current generation. The results helped us quantitatively understand why microbes growing slowly have the potential to make good use of fuel in MFCs. At the same time, slow growth does not necessarily result in speedy respiration. Alternative respirations may exist under the same growth state due to redundant pathways in the metabolic network. The big difference between the maximum and minimum respiration mainly results from the total formate secretion. With iterative flux variability analysis, a relatively ideal model of variant of *G. sulfurreducens* PCA was reconstructed by deleting several enzymes in the wild model, which could reach simultaneous suboptimal growth and maximum respiration. Under this ideal condition, flux towards extracellular electron transfer rather than for biosynthesis is beneficial for the conversion of organic matter to electricity without large accumulations of biomass and electricigens may maximize utilization of limited fuel. Our simulations will provide an insight into the enhanced current-generating mechanism and identify theoretical range of respiration rates for guiding strain improvement in MFCs.

## Introduction

Microbial Fuel Cells [1,2] are devices that convert a diverse range of organic matters to electricity with microbes serving as catalysts. As issues on energy resources inadequacy and environmental pollution are growing, MFCs, which exhibit unique working mechanism, have gained increasing attention in the bioenergy field. Practical applications of MFCs are fascinating, including power production from waste water combined with wastewater treatment, oxidation of contaminants to harmless carbon dioxide using an electrode as the electron acceptor, reduction of toxic metals to insoluble forms with an electrode as the electron donor, and driving small-scale portable electronics, microrobots, and so on. On the other hand, widespread utilization of MFCs cannot be expected because of the current bottleneck in power production and costing materials. Recently, the conductive biofilms of *Geobacter sulfurreducens* have been utilized to enhance the capacity for current production [3].

The activity of electricigens [4,5] that performs anaerobic respiration is an essential requirement in MFC systems. Electricigens can completely oxidize organic matters, resulting in extracellular electron transfer to anodes via the entire respiratory chain, and then to cathodes via the external electric circuit to reduce terminal electron acceptors, such as O_2_、Mn^4+^. Unsurprisingly, direct correlation exists among current production, extracellular electron transfer and oxidative dissimilation of electricigens to generate electrons. The stronger oxidative dissimilation is, the faster extracellular electron transfer is, and consequently more power is produced.

Under natural conditions where there are short of evolutionary pressure on electricigens to oxidize electron donors rapidly, electricigens tend to maximize growth companied by slow electron generation. Anodes of MFCs are likely to favor slow-growing, also called suboptimal-growing microbes, rather than microbes capable of oxidizing a great deal of fuel [4,5,6]. Exerting selective pressure for faster electron donor oxidization on microbes is good for respiration, but little knowledge is known about metabolism of mutant strains other than enhanced extracellular electron transfer [7,8,9,10].

Cellular metabolism state is governed by metabolic flux distribution. Investigating the metabolic process quantitatively at a system level enables strain improvement [11,12]. Although measurement of the metabolic flux at a genome scale using experimental methods is feasible, this process consume a lot of time and labor costs. As a wealth of complete genome sequences and genome annotation tools become available, efforts have been exerted on reconstructing *in silico* models of biological systems, and subsequently simulating the cellular processes employing these computational models, providing biologists with thorough insights into cellular behaviors [13,14].

*Geobacter* species have the ability to oxidize completely a variety of organic compounds to carbon dioxide under anaerobic conditions coupled with electron transfer outside the cell over long distances [15,16]. The ability makes them excellent models for exploring physiological capabilities of microorganisms present in a diverse range of sedimentary environments. In this work, we employed *in silico* algorithms to simulate different current-generating metabolic states with a fixed fuel uptake rate, and reconstructed a relatively ideal model of variant of *G. sulfurreducens* PCA. The results enabled us to quantitatively understand the enhanced current-generating mechanism and develop electricigens that utilize electron donors efficiently.

## Materials and Methods

### The metabolic model of *G. sulfurreducens* PCA

The *in silico* model of *G. sulfurreducens* PCA, a model reconstructed in 2005 and updated in 2008 using the SimPheny (Genomatica, San Diego, CA) platform was applied in the following simulations [17,18]. The compartmentalized model contained 747genes, 698 metabolites, and 649 reactions. All reactions occurred in either the cytoplasm or the extracellular space. In the 649 reactions, 530 metabolic reactions were obtained based on sequence similarity, available biochemical databases (eg KEGG, BioCyc, etc.) and genetic evidences. In terms of available substrates and *G. sulfurreducens* secretory products, 64 reactions corresponding to transport between the cytoplasm and extracellular space were added. The rest were exchange reactions, which allowed extracellular metabolites to enter into or end products to be secreted from the cell.

The metabolic reconstruction used electron donor acetate as carbon source and energy source simultaneously, with soluble fumarate or insoluble Fe^3+^ as the electron acceptor. Biomass reaction agg_GS13m_2, which represents growth-associated biosynthetic demands, contains biomass components and essential growth metabolites, and the flux through it is equal to the strain growth rate *v*_gr_. *G. sulfurreducens* could completely oxidize acetate to carbon dioxide under anaerobic conditions, together with electron transfer outside the cell to reduce Fe^3+^. The flux through exchange reaction EX_fe3(e) is equal to the rate of Fe^3+^ reduction, also named as respiration rate *v*_res_, and its value indicates the speed of extracellular electron transfer. Both the anode of MFC systems and Fe^3+^ are insoluble electron acceptors that can be reduced by *G. sulfurreducens* with a similar mechanism, hence, Fe^3+^ was taken as the electron acceptor to simulate cellular metabolism in MFCs [17].

### Flux Balance Analysis and Flux Variability Analysis

Flux Balance Analysis (FBA) is a linear programming approach, an approach assuming the metabolic network at steady state to ensure the rate at which every metabolite is consumed is equal to that of being produced. FBA allows predictive computation of the cellular flux distribution that maximizes objective function with a particular meaning under certain cultivation conditions [19]. The form of an FBA problem concerning a metabolic model with *m* metabolites and *n* reactions is given below:


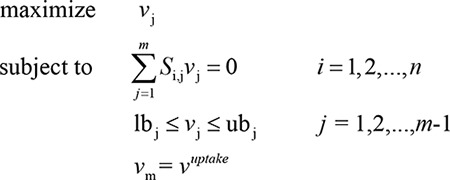
(1)

Parameters description: *S*_*i*,*j*_ is the stoichiometric coefficient of metabolite *i* in reaction*j*, *v*_*j*_  is the flux through reaction*j*, lb_j_ is the minimum allowable flux through reaction*j*, ub_j_ is the maximum allowable flux through reaction*j*, and $v_{}^{uptake}\;$ is the flux through exchange reaction regarding carbon source and energy source.

Flux Variability Analysis (FVA) [20] has the same principles as FBA except for the growth rate constraint, which can be used to study the entire range of theoretically achievable respiration rate. Here we register the subscript of *v*_*gr*_ as*m*−1, and the following two formulations was applied to examine alternate respiration states *v*_*res*_：


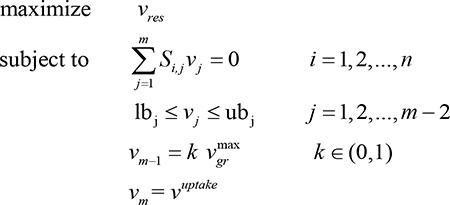
(2)


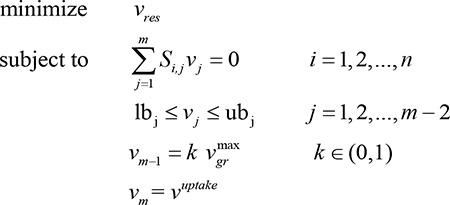
(3)

### Simulation of current-generating metabolism

*G. sulfurreducens* can use acetate as carbon source and energy source. When simulating optimal growth using Flux Balance Analysis (FBA) [19], acetate uptake rate *v* ^uptake^ was set as a fixed value, and the rate of biomass reaction agg_GS13m_2, *v*_gr_, was defined as objective function with the need to be maximized.

If microbes grow suboptimally, the growth rate is less than that of optimal growth. The equation *v*_gr_ =*k*﹒*v*_gr_^max^,*k*∈(0,1)   could be employed to describe the suboptimal growth state, where *v*_gr_^max^ is the optimal value for growth. Flux Variability Analysis (FVA) [20] was carried out to identify the maximum and minimum respiratory rate under one same suboptimal growth state. All above problems were solved using COBRA (Constraints Based Reconstruction and Analysis) toolbox accessed via MATLAB modeling environment [21].

## Results and Discussion

### Competitive relationship between oxidative respiration and cell growth

**Figure 1:**
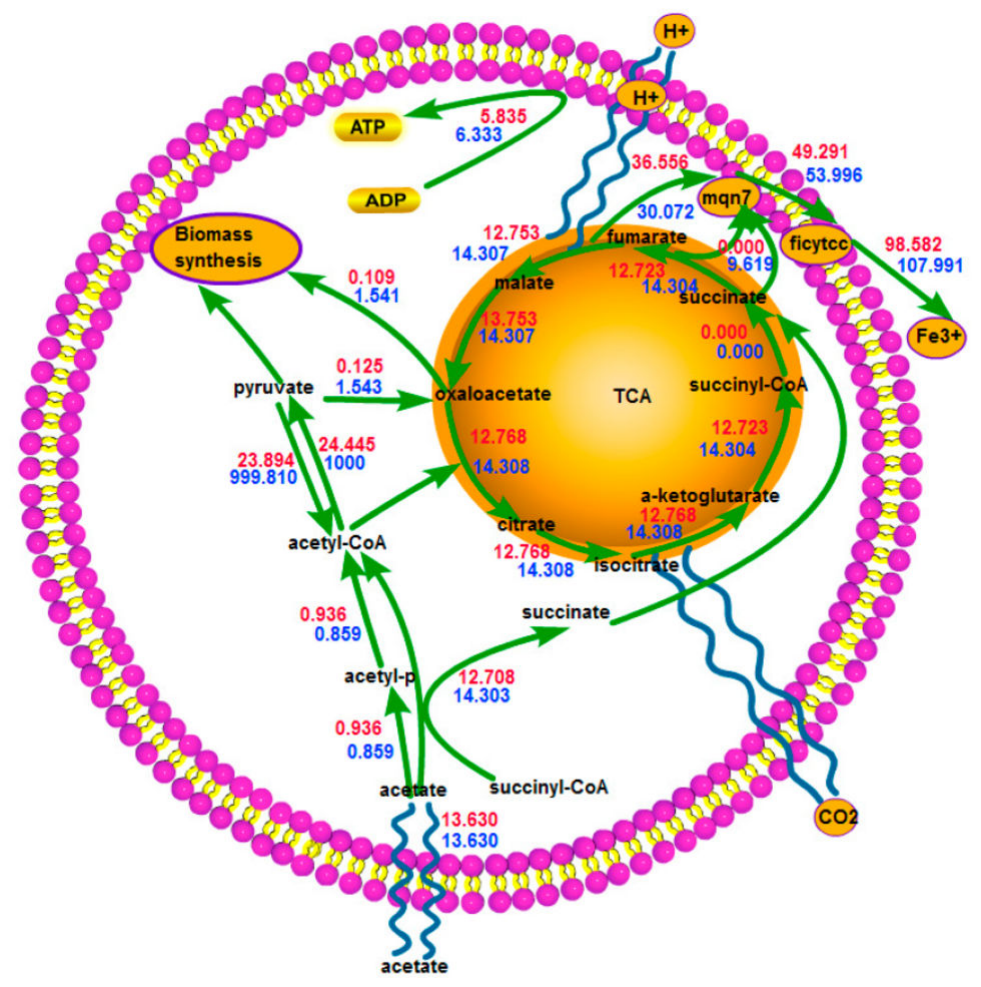
Central metabolism of *G. sulfurreducens* and simulation results of optimal and suboptimal growth. Red values for optimal and blue values for suboptimal growth.

In order to examine the impact of growth state on the current production capacity, we simulated optimal and suboptimal growth by constraining acetate uptake rate at 13.630mmol/g.dw/h. We confined our attention to central metabolism (see Figure **1**), a coupled characterization of metabolic pathways from extending previous computational and experimental analysis of the metabolic model of *G. sulfurreducens* [15,22]. Simulation results of optimal and suboptimal growth were placed in Figure **1**.

From the conlums of *v*_gr_ and *v*_res_ of Table **1**, with the increasing of *v*_gr_, *v*_res_ would decreased. The result indicated that there may be a competitive relationship between oxidative respiration and fuel assimilation into biomass. So microbes growing suboptimally could make good use of fuel for current generation in MFC systems.

**Table 1:** Predicted the entire range of respiratory rates of *G. sulfurreducens*during *in silico* optimal and suboptimal growth with acetate uptake rate at 13.630mmol/g.dw/h.

*v*_gr_(h^-1^)	*v*_res_(mmol/g.dw/h)	
	minimum	maximum
0.060	98.447	98.582
0.054	94.633	99.603
0.048	90.576	100.651
0.042	86.519	101.700
0.036	82.462	102.749
0.030	78.405	103.797
0.024	74.348	104.846
0.018	70.291	105.894
0.012	66.234	106.943
0.006	62.177	107.991
0.000	58.120	109.040

After being transported into the cell, acetate was activated to acetyl-CoA via acetate kinase followed by acetyl-CoA transferase [15]. Over 90% of acetyl-CoA was then directed to the TCA cycle for carbon dioxide, NADH, NADPH and reduced ferredoxin generation. The rest was used for fatty acid metabolism, amino acid metabolism and pyruvate synthesis. Pyruvate had a dual role in phosphoenolpyruvate (PEP) synthesis for gluconeogenesis and anapleurotic reaction, a reaction used for converting pyruvate to oxaloacetate via pyruvate carboxylase. Anapleurotic reaction was the process of replenishing the TCA cycle intermediate oxaloacetate - a small part of which was provided to synthesize PEP and the biomass precursor aspartate, which ensured that the TCA cycle ran smoothly and continuously [22].

ATP production of *G. sulfurreducens* was completely dependent on electrogenic electron transport. Electrons carried in reducing equivalents were transferred into the inner membrane via NADH or NADPH dehydrogenase, and protons were pumped out of the cytoplasm for ATP synthesis via ATP synthase. Although it was well known that c-type cytochromes were important for extracellular electron transfer, a specific electron transfer chain for electron transfer out of the inner membrane to Fe^3+^ or electrodes had not been determined for *G. sulfurreducens* [23]. In the model of *G. sulfurreducens*, a simplified extracellular electron transfer process was developed by NADH or NADPH dehydrogenase, cytochrome-c reductase and then Fe^3+^ reductase.

Simulation results presented here indicated that when microbes grew suboptimally, more fuel was directed to the TCA cycle to generate electron and energy, resulting in less being used for biomass synthesis compared with that optimal growth. Therefore microbes growing suboptimally may make good use of fuel for current generation in MFC systems.

### Effect of alternative flux distributions on the respiration of *G. sulfurreducens*

Computational results presented in the above section indicated that suboptimal growth was good for the improvement of respiration rate. However, alternative respiratory states may exist under the same growth state as a result of the inherent redundancies built into metabolic networks [20]. FVA was used to characterize the entire range of respiratory states (Table **1**).

**Figure 2:**
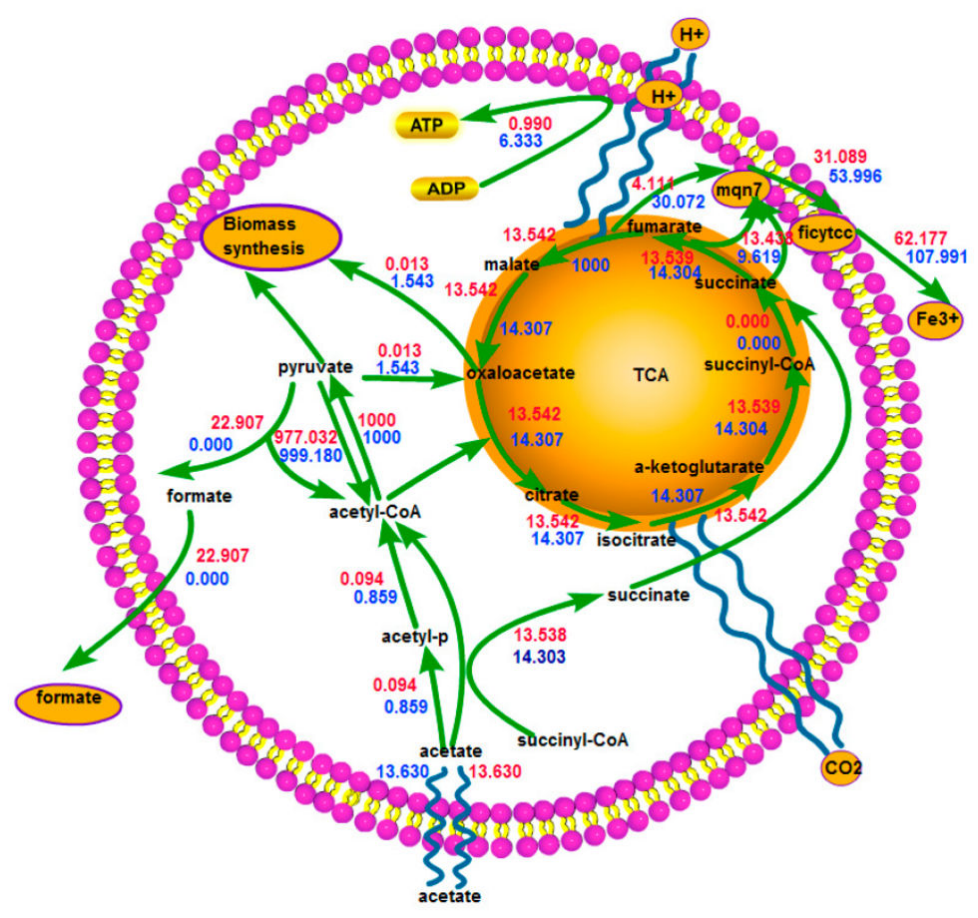
Alternative flux distributions through central metabolism of *G. sulfurreducens* during *in silico* growth at suboptimal growth of 0.006h^-1^. Predicted flux distributions through central metabolism in *G. sulfurreducens* during *in* *silico* suboptimal growth. Red values indicated the flux distribution corresponding to *in* *silico* minimum respiration at the rate of 62.177mmol/g.dw/h, and blue values corresponded to *in* *silico* maximum respiration at the rate of 107.991 mmol/g.dw/h. Alternative respiration was simulated by constraining the growth rate at 0.006h^-1^and maximizing/minimizing the respiration rate.

The genome-scale flux distributions were examined to illustrate the respiratory rate variance between the maximum and minimum respiration corresponding to all simulated growth states (from 0% to 90%). Excluding the reactions whose flux variances were less than 1% of their original flux values, those reactions whose flux distributions existed remarkable variance (larger than 1% of their original flux values) included five metabolic subsystems: central metabolism, transport, energy metabolism, amino acid metabolism, and exchange. Because of the same reason leading to the respiratory rate variance, we only paid attention to the suboptimal growth rate of 0.006h^-1^, a value that was 10% of that of optimal growth, and we thought this may be the best circumstance for electron extraction from the substrate. Figure **2** has illustrated alternative flux distributions through central metabolism of *G. sulfurreducens*, while Table **S1** in the supplemental materials provided details on all the reactions whose fluxes exist variance (The supplementary information of reference [18] have provided a look-up table for the whole names of all the reactions and metabolites).

Interconversion from acetyl-CoA to pyruvate, the first step of assimilation in *G. sulfurreducens*, had three pathways (pyruvate dehydrogenase, formate c-acetyltransferase, pyruvate synthase). With regard to maximum respiration, only two pathways were activated and no formate was produced. In contrast, regarding minimum respiration, three pathways were all activated and formate was produced at a rate of 22.907mmol/g.dw/h. Formate was chosen to be secreted as an end product outside the cell totally via proton symport instead of oxidation via formate dehydrogenase, resulting in the loss of electrons. At the same time, less acetate (13.544 vs 14.309 mmol/g.dw/h) was directed toward the TCA cycle, from which the majority of electrons were derived for *G. sulfurreducens* [24]. The conclusion could be arrived that under minimum respiration, less acetate towards TCA cycle and the total secretion of formate caused electron loss and subsequent large respiration rate difference from maximum respiration.

Cell need for energy was crucial to metabolism regulation. Increased cellular energy consumption inspired reducing equivalent-producing reactions to increase their rates to meet more energy demand [25]. We extracted reducing equivalent-producing reactions in central metabolism or amino acid metabolism in Table **S2** for Table **S1** in the supplemental materials. Of all 8 reducing equivalent-producing reactions, 5 reactions were involved in the TCA cycle and the redundant pathway for conversion of pyruvate to acetyl-CoA. The eight reactions had higher metabolic fluxes under maximum respiration. Especially, conversion of pyruvate to acetyl-CoA had the greatest difference (999.180 vs 977.038 mmol/g.dw/h.). Subsequently fluxes through the simplified extracellular electron transfer chain (NADH or NADPH dehydrogenase, cytochrome-c reductase and Fe^3+^ reductase) and ATP synthetic reaction (6.333 vs 0.990 mmol/g.dw/h) also increased. Unexpectedly the flux through NADPH dehydrogenase reaction was predicted to decrease (9.619 vs 13.438 mmol/g.dw/h). Conversion of pyruvate to acetyl-CoA, one of the redundancies in central metabolism of *G. sulfurreducens* [22], contributed largely to the cell for reducing equivalent production discrepancy and consequent respiration discrepancy. The result also suggested that pyruvate dehydrogenase cannot be substituted by formate c-acetyltransferase or pyruvate synthase and different enzymes presented for different tasks [17].

The discrepancy between maximum and minimum respiration increased along with the decrease of growth rate (Table **1**). We have investigated the suboptimal growth rate of 0.054h^-1^, a value that was 90% of that of optimal growth. The flux difference through pyruvate dehydrogenase reaction was 2.389 mmol/g.dw/h. (999.406 vs 997.017 mmol/g.dw/h) between maximum and minimum respiration. The value increased to 22.142 mmol/g.dw/h. (999.180 vs 977.038 mmol/g.dw/h) at a growth rate of 0.006 h^-1^. It was clear that the discrepancy between maximum and minimum respiration depended on the flux difference through reducing equivalent-producing reactions.

### Reconstruction of a relatively ideal model of variant of *G. sulfurreducens*

In the above section, an ideal current-generating metabolism state was given. The ideal state consisted of simultaneous suboptimal growth and maximum respiration. Under this ideal condition, flux towards extracellular electron transfer rather than for biosynthesis is beneficial for the conversion of organic matter to electricity without large accumulations of biomass that might otherwise plug specific parts of an aquifer. And at the same time, electricigens may maximize utilization of limited fuel.

The *in silico* metabolic model predicted that when *G. sulfurreducens* grew suboptimally at a rate of 0.006h^-1^, minimum respiratory rate was 62.177mmol/g.dw/h and caused by formate secretion. We deleted formate enzyme GSU0234 by constraining the flux through the reaction catalyzed by it at 0 mmol/g.dw/h, and after FVA, minimum respiratory rate increased to 75.267 mmol/g.dw/h, which was caused by pyruvate secretion. Then enzymes for pyruvate, L-valine, L-leucine, citrate, L-isoleucine, L-proline, L-lysine, L-cysteine, h2 and n2 were determined through iterative FVA and were deleted one by one. A relatively ideal model of variant for *G. sulfurreducens* strain was reconstructed finally by modifying the wild metabolic model in this way (Table **2**). The modified model predicted that under suboptimal growth of 0.006h^-1^ condition, minimum respiratory rate increased to 107.991 mmol/g.dw/h, a value that was equal to that of maximum respiration. Of all the simulated variants, formate-deficient variant has the biggest discrepancy between minimum and maximum respiration. The relatively ideal variant model was also validated with different suboptimal growth.

**Table 2:** Predicted the entire range of respiratory rate of variants of *G. sulfurreducens* during *in silico* suboptimal growth of 0.006h^-1^ with acetate uptake rate at 13.630mmol/g.dw/h.

Reactions (Enzymes) knockout	*v*_res_(mmol/g.dw/h)	
	minimum	maximum
FORt2	75.267	107.991
FORt2+ PYRt2	75.652	107.991
FORt2+ PYRt2+ VALt6	76.754	107.991
FORt2+ PYRt2+ VALt6+ LEUabc	86.290	107.991
FORt2+ PYRt2+ VALt6+ LEUabc+ CITt6	86.516	107.991
FORt2+ PYRt2+ VALt6+ LEUabc+ CITt6+ILEabc	87.561	107.991
FORt2+ PYRt2+ VALt6+ LEUabc+ CITt6+ILEabc + PROt5	89.127	107.991
FORt2+ PYRt2+ VALt6+ LEUabc+ CITt6+ILEabc + PROt5+ LYSt3	97.141	107.991
FORt2+ PYRt2+ VALt6+ LEUabc+ CITt6+ILEabc + PROt5+ LYSt3+CYSabc	107.828	107.991
FORt2+ PYRt2+ VALt6+ LEUabc+ CITt6+ILEabc + PROt5+ LYSt3+CYSabc+ N2t+ H2td	107.991	107.991

We examined the full range of flux through every reaction in the ideal modified model corresponding to the suboptimal growth of 0.006h^-1^ with acetate uptake rate in 13.630 mmol/g.dw/h (Table **S3** has provided the detail). Of all 705 reactions, 412 reactions were non-functional, that is, deletion of corresponding reactions had no impact on current-generating metabolism of the ideal variant of *G. sulfurreducens*. These reactions were involved in almost every subsystem. 101 reactions occurred at a constant rate.

**Figure 3:**
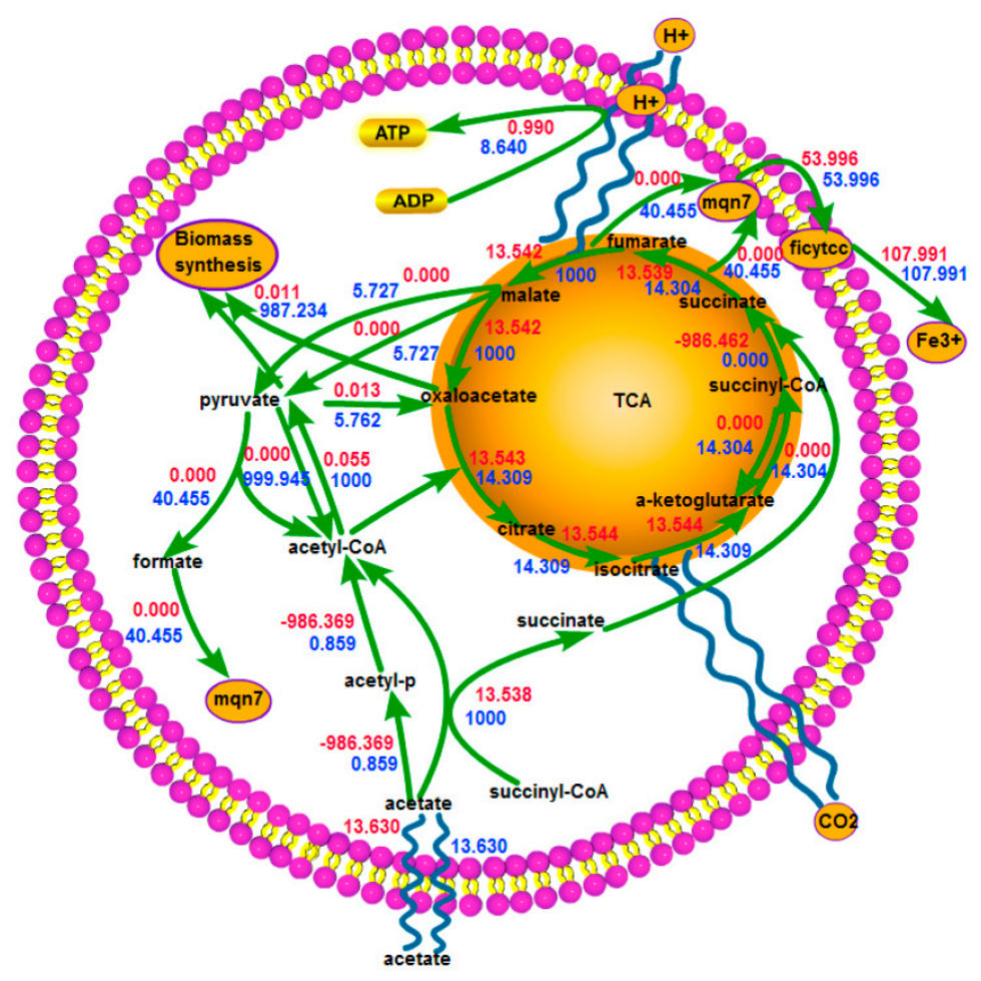
Flux distributions through central metabolism of a relatively ideal variant model of *G. sulfurreducens* during *in silico* growth at suboptimal growth of 0.006h^-1^. Predicted flux distributions through central metabolism in the ideal variant of *G. sulfurreducens* during *in* *silico* growth. Red values indicated the flux distribution corresponding to *in* *silico* minimum energy synthesis at the rate of 0.990 mmol/g.dw/h, and blue values corresponded to *in* *silico* maximum energy synthesis at the rate of 8.640 mmol/g.dw/h. Alternative energy synthesis was simulated by constraining the growth rate at 0.006 h^-1^ and maximizing/minimizing the energy synthesis rate.

As shown in Figure **3**, the variant had two pathways for conversion of a-ketoglutarate to succinyl-CoA: a-ketoglutarate dehydrogenase and a-ketoglutarate synthase. The latter could produce reduced ferredoxin. All fluxes through the other reactions in TCA cycle were variable [22]. Both malate and oxaloacetate could be produced at a predicted rate of 1000 mmol/g.dw/h. Conversion of succinyl-CoA to succinate via acetate-CoA transferase may not be the sole pathway, since the model predicted that maximum flux through the reaction catalyzed by succinyl-CoA synthetase for conversion of succinyl-CoA to succinate was 986.462 mmol/g.dw/h instead of 0 mmol/g.dw/h. Pyruvate was able to be produced via malic enzyme (NAD) or malic enzyme (NADP) at a rate of 5.727 mmol/g.dw/h.

Examination of the energy metabolism subsystem in the modified model found that owing to deletion of formate transport enzyme, formate produced via formate c-acetyltransferase was only consumed via formate dehydrogenase at a maximum rate of 40.455 mmol/g.dw/h. Then menaquinol reduced via formate dehydrogenase or NADH or NADPH dehydrogenase was oxidized via cytochrome-c reductase at a constant rate of 53.996 mmol/g.dw/h and subsequent Fe^3+^ reduced at a constant rate of 107.991 mmol/g.dw/h. The first step of the simplified extracellular electron transfer chain of the ideal variant was expanded to involve NADH dehydrogenase, NADPH dehydrogenase and formate dehydrogenase.

The flux through ATP synthetic reaction was variable. Maximum and minimum fluxes were 8.640 and 0.990 mmol/g.dw/h respectively. We further investigated the full flux range through ATP synthetic reaction corresponding to different growth (Table **3**). Increasing cellular need for energy was neither the sufficient condition nor the necessary condition for respiration improvement [25]. When the variant grew at a rate of 0.018h^-1^ and respired at a rate of 105.894 mmol/g.dw/h, energy could be produced at a minimum rate of 2.070 mmol/g.dw/h and its rate may increase owing to the increasing rate of futile cycle for consumption of energy. When the variant grew at a rate of 0.006 h^-1^ and respired at a rate of 107.991 mmol/g.dw/h, energy could be produced at a minimum rate of 0.990 mmol/g.dw/h with no futile cycle for energy consumption.

**Table 3:** Predicted the entire flux range through ATP synthetic reaction of a ideal variant of *G. sulfurreducens* corresponding to different *in silico* growth with acetate uptake rate at 13.630mmol/g.dw/h.

*v*_gr_(h^-1^)	*v*_ATP_(mmol/g.dw/h)	
	minimum	maximum
0.060	5.850	5.967
0.054	5.310	6.264
0.048	4.770	6.561
0.042	4.230	6.858
0.036	3.690	7.155
0.030	3.150	7.452
0.024	2.610	7.749
0.018	2.070	8.046
0.012	1.530	8.343
0.006	0.990	8.640
0.000	0.450	8.640

We have searched all the literatures concerning *G. sulfurreducens* with paying special attention to the strain modification, but did not find some modification strategies which were similar with what we have put forward (Table **2**), so we think our strategy for *G. sulfurreducens* modification is new. In the next stage, we will do experiments of strain modification for the final variant of *G. sulfurreducens*, i.e. deleting “FORt2 + PYRt2 + VALt6 + LEUabc + CITt6 + ILEabc + PROt5 + LYSt3 + CYSabc + N2t + H2td”, to verify our *in silico* ideal mutant for *G. sulfurreducens* strain.

## Conclusion

How to increase the respiration rate has been catching great attention in the MFC field. Increased rates of respiration of electricigens indicated it is possible to compel microbes to evolve towards enhanced current generation with appropriate selective pressure. However, we know little about enhanced current-generating mechanism and the feasible range of respiratory rate under one growth state.

In this paper, we applied *in silico* simulation algorithms of FBA to the metabolic network model of *G. sulfurreducens* to simulate various current-generating metabolism states with a certain uptake rate of electron donor acetate. we found that when *G. sulfurreducens* grows suboptimally, more substrate is completely oxidized to generate electrons, resulting in a higher respiration.

Alternative respirations exist regarding one same suboptimal growth because of built-in metabolic network redundancies. Formate secreting outside the cell is a major reason for the big difference between maximum and minimum respiration. Through FVA method, we found that simultaneous suboptimal growth and maximum respiration is the desired current-generating metabolism state.

Through iterative FVA, a relatively ideal metabolic model was reconstructed by deletion of several enzymes. Increasing cellular need for energy doesn’t necessarily mean respiration improvement, and vice versa because of existence of futile cycle for energy consumption.

These studies indicated developing microbes to make them grow suboptimally is just the first step for microbe improvement. Multiple rounds of strain modification are needed until the metabolic flux distribution agrees with or is close to that of *in silico* simulation related to ideal current-generating metabolism.

## Supporting Information

Table S1 **This table provides information on all the reactions whose fluxes exist variance.**
(XLS)

Table S2 **This table provides information on all reducing equivalent-producing reactions.**
(XLS)

Table S3 **This table provides information on the full flux range through every reaction.**
(XLS)

## References

[1] Franks AE, Nevin KP (2010) Microbial fuel cells, a current review. Energies 3: 899-919. doi:10.3390/en3050899.

[2] Franks AE, Malvankar N, Nevin KP (2010) Bacterial biofilms: the powerhouse of a microbial fuel cell. Biofuels 1: 589-604. doi:10.4155/bfs.10.25.

[3] Lovley DR (2006) Bug juice: harvesting electricity with microorganisms. Nat Rev Microbiol 4: 497-508. doi:10.1038/nrmicro1442. PubMed: 16778836. 16778836 10.1038/nrmicro1442

[4] Ching L, Nikhil SM, Ashley EF et al. (2013) Engineering geobacter sulfurreducens to produce a highly cohesive conductive matrix with enhanced capacity for current production. Energy. J Environ Sci 6: 1901-1908.

[5] Logan BE (2009) Exoelectrogenic bacteria that power microbial fuel cells. Nat Rev Microbiol 7: 375-381. doi:10.1038/nrmicro2113. PubMed: 19330018. 19330018 10.1038/nrmicro2113

[6] Lovley DR (2006) Taming electricigens. Scientist 20: 46-46.

[7] Strycharz SM, Malanoski AP, Snider RM, Yi H, Lovely DR et al. (2011) Application of cyclic voltammetry to investigate enhanced catalytic current generation by biofilm-modified anodes of Geobacter sulfurreducens strain DL1 vs. variant strain KN400. Energy. J Environ Sci 4: 896-913.

[8] Yi H, Nevin KP, Kim BC, Franks AE, Klimes A et al. (2009) Selection of a variant of Geobacter sulfurreducens with enhanced capacity for current production in microbial fuel cells. Biosens Bioelectron 24: 3498-3503. doi:10.1016/j.bios.2009.05.004. PubMed: 19487117. 19487117 10.1016/j.bios.2009.05.004

[9] Tremblay PL, Summers ZM, Glaven RH, Nevin KP, Zengler K et al. (2011) A c-type cytochrome and a transcriptional regulator responsible for enhanced extracellular electron transfer in Geobacter sulfurreducens revealed by adaptive evolution. Environ Microbiol 13: 13-23. doi:10.1111/j.1462-2920.2010.02302.x. PubMed: 20636372. 20636372 10.1111/j.1462-2920.2010.02302.x

[10] Inoue K, Qian X, Morgado L, Kim BC, Mester T et al. (2010) Purification and characterization of OmcZ, an outer-surface, octaheme c-type cytochrome essential for optimal current production by Geobacter sulfurreducens. Appl Environ Microbiol 76: 3999-4007. doi:10.1128/AEM.00027-10. PubMed: 20400562. 20400562 10.1128/AEM.00027-10 PMC2893489

[11] Park JH, Lee SY, Kim TY, Kim HU (2008) Application of systems biology for bioprocess development. Trends Biotechnol 26: 404-412. doi:10.1016/j.tibtech.2008.05.001. PubMed: 18582974. 18582974 10.1016/j.tibtech.2008.05.001

[12] Lee JM, Gianchandani EP, Papin JA (2006) Flux balance analysis in the era of metabolomics. Brief Bioinform 7: 140-150. doi:10.1093/bib/bbl007. PubMed: 16772264. 16772264 10.1093/bib/bbl007

[13] Thiele I, Palsson BØ (2010) A protocol for generating a high-quality genome-scale metabolic reconstruction. Nat Protoc 5: 93-121. doi:10.1038/nprot.2009.203. PubMed: 20057383. 20057383 10.1038/nprot.2009.203 PMC3125167

[14] Oberhardt MA, Palsson BØ, Papin JA (2009) Applications of genome-scale metabolic reconstructions. Mol Syst Biol 5: 320 PubMed: 19888215. 19888215 10.1038/msb.2009.77 PMC2795471

[15] Mahadevan R, Palsson BØ, Lovley DR (2010) In situ to in silico and back: elucidating the physiology and ecology of Geobacter spp. using genome-scale modelling. Nat Rev Microbiol 9: 39-50. PubMed: 21132020. 21132020 10.1038/nrmicro2456

[16] Malvankar NS, Tuominen MT, Lovley DR (2012) Biofilm conductivity is a decisive variable for high-current-density Geobacter sulfurreducens microbial fuel cells. Energy. J Environ Sci 5: 5790-5797.

[17] Mahadevan R, Bond DR, Butler JE, Esteve-Nuñez A, Coppi MV et al. (2006) Characterization of metabolism in the Fe (III)-reducing organism Geobacter sulfurreducens by constraint-based modeling. Appl Environ Microbiol 72: 1558-1568. doi:10.1128/AEM.72.2.1558-1568.2006. PubMed: 16461711. 16461711 10.1128/AEM.72.2.1558-1568.2006 PMC1392927

[18] Sun J, Sayyar B, Butler JE, Pharkya P, Fahland TR et al. (2009) Genome-scale constraint-based modeling of Geobacter metallireducens. BMC syst biol 3: 15. 19175927 10.1186/1752-0509-3-15 PMC2640342

[19] Orth JD, Thiele I, Palsson BØ (2010) What is flux balance analysis? Nat Biotechnol 28: 245-248. doi:10.1038/nbt.1614. PubMed: 20212490. 20212490 10.1038/nbt.1614 PMC3108565

[20] Mahadevan R, Schilling CH (2003) The effects of alternate optimal solutions in constraint-based genome-scale metabolic models. Metab Eng 5: 264-276. doi:10.1016/j.ymben.2003.09.002. PubMed: 14642354. 14642354 10.1016/j.ymben.2003.09.002

[21] Schellenberger J, Que R, Fleming RMT, Thiele I, Orth JD et al. (2011) Quantitative prediction of cellular metabolism with constraint-based models: the COBRA Toolbox v2. 0. Nat Protoc 6: 1290-1307. doi:10.1038/nprot.2011.308. PubMed: 21886097. 21886097 10.1038/nprot.2011.308 PMC3319681

[22] Segura D, Mahadevan R, Juárez K, Lovley DR (2008) Computational and experimental analysis of redundancy in the central metabolism of Geobacter sulfurreducens. PLOS Comput Biol 4: 36. doi:10.1371/journal.pcbi.0040036. PubMed: 18266464. 10.1371/journal.pcbi.0040036 PMC2233667 18266464

[23] Butler JE, Young ND, Lovley DR (2010) Evolution of electron transfer out of the cell: comparative genomics of six Geobacter genomes. BMC Genomics 11: 40. doi:10.1186/1471-2164-11-40. PubMed: 20078895. 20078895 10.1186/1471-2164-11-40 PMC2825233

[24] Galushko AS, Schink B (2000) Oxidation of acetate through reactions of the citric acid cycle by Geobacter sulfurreducens in pure culture and in syntrophic coculture. Arch Microbiol 174: 314-321. doi:10.1007/s002030000208. PubMed: 11131021. 11131021 10.1007/s002030000208

[25] Izallalen M, Mahadevan R, Burgard A, Postier B, Didonato R et al. (2008) Geobacter sulfurreducens strain engineered for increased rates of respiration. Metab Eng 10: 267-275. doi:10.1016/j.ymben.2008.06.005. PubMed: 18644460. 18644460 10.1016/j.ymben.2008.06.005

